# Genomic regions associated with heat stress tolerance in tropical maize (*Zea mays* L.)

**DOI:** 10.1038/s41598-021-93061-7

**Published:** 2021-07-02

**Authors:** Kaliyamoorthy Seetharam, Prakash H. Kuchanur, K. B. Koirala, Mahendra Prasad Tripathi, Ayyanagouda Patil, Viswanadh Sudarsanam, Reshmi Rani Das, Ramesh Chaurasia, Kamal Pandey, Hindu Vemuri, Madhumal Thayil Vinayan, Sudha K. Nair, Raman Babu, P. H. Zaidi

**Affiliations:** 1 Asian Regional Maize Program, International Maize and Wheat Improvement Center (CIMMYT), Patancheru, Hyderabad, 502 324 Telangana India; 2 University of Agricultural Sciences, Raichur, Karnataka India; 3 National Maize Research Program (NMRP), Rampur, Chitwan Nepal; 4 Kaveri Seeds Limited, Hyderabad, India; 5 Corteva Agrisciences, Hyderabad, India

**Keywords:** Genetics, Plant sciences

## Abstract

With progressive climate change and the associated increase in mean temperature, heat stress tolerance has emerged as one of the key traits in the product profile of the maize breeding pipeline for lowland tropics. The present study aims to identify the genomic regions associated with heat stress tolerance in tropical maize. An association mapping panel, called the heat tolerant association mapping (HTAM) panel, was constituted by involving a total of 543 tropical maize inbred lines from diverse genetic backgrounds, test-crossed and phenotyped across nine locations in South Asia under natural heat stress. The panel was genotyped using a genotyping-by-sequencing (GBS) platform. Considering the large variations in vapor pressure deficit (VPD) at high temperature (Tmax) across different phenotyping locations, genome-wide association study (GWAS) was conducted separately for each location. The individual location GWAS identified a total of 269 novel significant single nucleotide polymorphisms (SNPs) for grain yield under heat stress at a p value of < 10^–5^. A total of 175 SNPs were found in 140 unique gene models implicated in various biological pathway responses to different abiotic stresses. Haplotype trend regression (HTR) analysis of the significant SNPs identified 26 haplotype blocks and 96 single SNP variants significant across one to five locations. The genomic regions identified based on GWAS and HTR analysis considering genomic region x environment interactions are useful for breeding efforts aimed at developing heat stress resilient maize cultivars for current and future climatic conditions through marker-assisted introgression into elite genetic backgrounds and/or genome-wide selection.

## Introduction

In Asia region maize has emerged as the third most important crop after rice and wheat, with average annual growth rate of 3.1% in maize area during 1993–2013^1^. Though the growth in maize area resulted in 2.4–5.6% increases in production, maize productivity in this region remain low with high inter-annual variability. Climate change effects accounted for over 50% of the total year-to-year variation in maize yields^2^. In Asia region, maize is largely (~ 80%) grown as rainfed crop and therefore is highly exposed to weather extremes, including intermittent drought and heat stress. Thus, there is a need for fast-tracking the development and deployment of stress-resilient maize to cope-up with extremes weather events. Due to exponential increase in maize demand spring maize has emerged as third cropping season where the crop is grown during the hot-summer period of the year (Feb–May). Maize crop grown during spring season is invariably exposed to high temperature regimes (> 35 ℃) during most part of the critical crop growth period, especially from the late vegetative stage until early grain filling. Also, during the main maize cropping season (monsoon season), which accounts for over 70% of the total maize area in the region, there is an increased frequency of drought years along with high temperature that severely affects maize yields. Climate modeling studies suggested that increased day as well as night temperatures will become more common in the tropics and that may significantly affect food production^3,4^. South Asia region has been identified as one of the hot-spot regions for climate change effects; therefore, without sufficient adaptation measures, the region is likely to suffer negative impacts of the climate change^5^. During the past 2 decades, Asian tropics have experienced frequent episodes of extreme weather events including increased day/night temperatures, apart from scattered drought/heat stress periods almost every year in one or another country of South Asia^6^. Impact assessment of climate change effects on maize in South Asia showed that heat stress affected areas of South Asia will increase significantly under future climates, especially in the pre-monsoon season (spring) and in monsoon season^7^.

Being a tropical crop, maize can survive brief exposures to extreme temperatures (< 0 °C and > 40 °C), but prolonged exposure to temperatures above 35 °C is unfavorable for crop growth. The temperatures above 40 °C during the reproductive stage can cause irreversible physiological damage and severely affect grain yield^8,9^. In view of recent climate change patterns in the lowland tropics, heat stress alone and in combination with drought, has emerged as one of the major production constraints for maize in several maize-dependent countries^6,10^. Lobell and Burke^11^ showed that an increase of 2 °C temperature would result in a greater yield penalty in maize crop than a 20% decrease in precipitation. Due to high vapor pressure deficit (VPD) under heat stress, maize crop experience severe physiological drought due to increased atmospheric demand for moisture driven by high temperature and low humidity, which explains the relatively high yield penalties under heat stress compared to drought. Unless frequently irrigated to maintain high humidity within crop canopy and to offset the effect of high VPD associated physiological drought, crops face the compound effects of heat plus drought, which results in relatively higher yield losses. The intensity of heat stress is defined by the prevailing VPD, which is a function of maximum temperature (Tmax) and relative humidity at Tmax^12^. Varying degree of VPD results in strong genotype × environment interaction effects and therefore maize crops exposed to heat stress at different locations may respond differently, depending on the level of VPD at Tmax^13^. These studies highlight the need to incorporate heat stress resilience into maize cultivars grown in lowland tropics. Understanding the genetic architecture of heat stress tolerance by identifying the genomic regions conferring heat tolerance and developing marker-trait associations can accelerate the development of heat stress-resilient maize varieties through marker-assisted ingression of validated genomic regions into elite genetic backgrounds and/or genomic selection. In recent years, GWAS has been extensively used to decipher genomic regions associated with various agronomic traits, quality traits, a range of diseases, and traits associated with key abiotic stresses^14^. Genomic regions associated with key traits under heat stress have also been identified using association mapping studies^15,16^. However, limited studies are available on complex traits such as grain yield under heat stress in tropical maize. Genome-wide association mapping for combined drought and heat tolerance^17^ identified 6 significant haplotype associations for grain yield under combined drought and heat stress management on chromosome 4. The study also identified significant hot-spots for grain yield exclusively under heat stress on chromosome 8 (bin 8.05 and 10.03) and on chromosome 4 for combined heat and drought stress (bin 4.02–4.03 and bin4.04). Another mapping study for heat stress tolerance in sub-tropical maize^18^ identified 12 significant SNP associations for grain yield under heat stress on a panel of 662 DH lines with tropical origin. These SNPS were localized on chromosomes 1, 3, 6, 7 and 10 accounting for about 18% of the phenotypic variation. In addition, the study also revealed significant haplotype trait associations on chromosomes 1, 3, and 7.

With this rationale, the present study aims to identify superior alleles associated with heat stress tolerance using genome-wide association studies (GWAS) and haplotype trend regression (HTR) analysis, in tropical working germplasm maintained in the breeding program.

## Materials and methods

### Association mapping panel

A set of 534 maize inbred lines representing the wide genetic background of tropical maize were included in the association mapping panel, named as heat tolerant association mapping (HTAM) panel (Supple. Table-1). The lines involved in the panel included the selected 449 lines derived from the International Maize and Wheat Improvement Center (CIMMYT), 52 lines from the Maize and Millet Research Institute (MMRI), Pakistan, 23 tropicalized temperate lines from Purdue University, USA, and 10 lines from Kaveri Seeds, Hyderabad, India. The CIMMYT lines involved in the panel were derived from various pools and populations, including several biparental pedigree populations developed by the CIMMYT-Asia maize program for abiotic stress breeding, and selected lines from pools/populations from CIMMYT-Mexico, such as La Posta Sequia-C7 (tropical late white-dent), DTPY-C9 (drought tolerant population—yellow), DTPW-C9 (drought tolerant population—white), G18 Sequia C5 (drought tolerant early yellow tropical population) and Pool16 BN Sequia-C5 (drought tolerant early white tropical population), which had reasonably good adaptation to the Asian tropics. Further details about the pools/populations from CIMMYT’s lowland tropical maize program can be found elsewhere^19^.

### Phenotyping under managed heat stress

The HTAM panel was test-crossed with an established tester line from CIMMYT (CML451) with high general combining ability. CML451 is a high yielding line widely used by lowland tropical breeding programs in Asia; it has good local adaptation and resistance to foliar diseases common in the region but is highly susceptible to heat stress. The test-cross progenies were phenotyped at nine locations during the spring season (March to June) of 2013 and 2014. In spring 2013, the testcrosses were evaluated in four locations of India and Nepal, viz., Raichur (RA) and B’gudi (BG-1), Karnataka and Ludhiana (LU), Punjab, India, and Nepalgunj (NG), Nepal. In spring 2014, the testcross were evaluated at five locations in India, viz.*,* Hyderabad (HY) and Baijenki (BJ) in Telangana state, at two locations in Jalandhar district (JL-1, JL-2) of Punjab and B’gudi (BG-2) in Karnataka. Locations were carefully selected based on weather data during the spring season (Feb–June) over the past 10 years, which showed that at all these locations, maximum temperature (Tmax) is usually > 35 °C after mid-March and invariably reaches close to 40 ℃ or more during the first fortnight of the month of May, and similar weather pattern was observed during the multilocation trials conducted in the year 2013 and 2014 (Supple. Table 2). The geographical coordinates of the trial locations are given in Table 1.

**Table 1:** Details of the locations and descriptive statistics for grain yield (t ha^−1^) of HTAM panel testcrosses evaluated under heat stress.

Locations	Latitude and longitude	Year	Entries	h^2^	Mean	Min	Max	LSD (5%)	σ^2^g	σ^2^e	F-Value
B'Gudi-1	16°.73′N; 76°.79′E	2013	290	0.62	2.77	1.26	4.36	1.86	0.74***	0.90	1.88
Ludhiana	30°.99′N; 75°.74′E	2013	290	0.50	3.61	1.99	5.26	2.38	0.75**	1.48	1.54
Nepalgunj	28°.05′N; 81°.61′E	2013	290	0.36	2.25	1.54	3.22	1.60	0.19***	0.67	1.30
Raichur	16°.22′N; 77°.38′E	2013	335	0.44	2.48	1.71	3.60	2.41	0.39***	1.51	1.27
Beijenki	18°.25′N; 79°.02′E	2014	420	0.53	2.56	1.15	4.87	2.32	0.80***	1.40	1.60
B'Gudi-2	16°.73′N; 76°.79′E	2014	285	0.69	3.19	1.27	5.49	2.23	1.43***	1.30	2.18
Hyderabad	17°.51′N; 78°.27′E	2014	479	0.61	3.65	2.20	5.24	1.72	0.61***	0.77	1.84
Jalandhar-1	31°.32′N; 75°.57′E	2014	471	0.49	4.63	3.23	5.51	2.03	0.35***	1.08	1.34
Jalandhar-2	31°.32′N; 75°.57′E	2014	435	0.41	6.81	5.42	8.06	2.53	0.59***	1.67	1.37

*h*^*2*^ broad-sense heritability, *LSD* least significant difference, *σ*^*2*^*g* genotypic variance, *σ*^*2*^*e* error variance.

***^,^**^,^*Significant at p < 0.001, 0.01 and 0.05.

Trials at all locations in both years were planted during the second or third week of March, except at the Jallandhar location, where one set was planted in the last week of February and the other set in the second week of March 2014. Spring season planting time at all the locations was adjusted in such a way that most of the reproductive stage, including flowering, pollination, fertilization, and early grain filling, is exposed to severe heat stress. The weather parameters, viz., maximum, minimum temperature, relative humidity and vapor pressure deficit (VPD), of the trial locations were captured for the cropping period (Supple. Table 2). All the trials were planted using an alpha-lattice design with two replications in rows of 4.0 m in length with 0.75 m row-to-row spacing and 0.2 m plant-to-plant spacing. At maturity, ears were harvested separately from each plot and the grain yield (GY) was recorded on a per plot basis. Final grain yield was calculated after adjusting the moisture content at 12.5% for each plot and converting it to tons per hectare (t ha^−1^).

### Phenotyping data analysis

Analyses of variance (ANOVA) for GY at individual locations were carried out using META-R (Multi-Environment Trial Analysis in R) software. Genetic (*σ*^*2*^*g*) and error (*σ*^*2*^*e*) variance components are estimated from ANOVA. Broad-sense heritability (*h*^*2*^) for individual locations was estimated as:

$${h}^{2}={\sigma }^{2}g/{\sigma }^{2}p$$

where *h*^*2*^ is the broad-sense heritability, *σ*^*2*^*g* is the genetic variance, *σ*^*2*^*p* is the phenotypic variance estimated as *σ*^*2*^*p* = *σ*^*2*^*g* + (*σ*^*2*^*e/r*)*,* where *σ*^*2*^*e* is the error variance and* r* is number of replications.

Best linear unbiased estimators (BLUEs) were estimated for individual locations as:

$${\text{Y}}_{\text{bir}}{=\mu +}{\text{G}}_{\text{i}}+{\text{R}}_{\text{r}}+{\text{B}}_{\text{br}}+{\text{e}}_{\text{bir}}$$

where *Y*_*bir*_ was the adjusted phenotypic observation for the *i*th genotype in the *b*th block within the *r*th replication. *µ* denoted the mean*, G*_*i*_ denoted the fixed effect of the *i*^*th*^ genotype and *e*_*bir*_ the residual error term. Descriptive statistics including mean, minimum, maximum and least significant difference (LSD) were also generated using standard procedures implemented in METAR. Best linear unbiased estimators (BLUEs) were used for GWAS.

### HTAM panel genotyping

DNA extraction was done following the modified CTAB method (CIMMYT Applied Molecular Genetics Laboratory, 2003), from 3 to 4 week young leaves of each inbred line in the HTAM panel raised for test-cross development. Single nucleotide polymorphisms (SNPs) were generated through GBS v2.7 using Illumina Hi-seq 2000/2500 at the Institute for Genomic Diversity, Cornell University, Ithaca, NY, USA. The physical coordinates of GBS SNPs were derived from AGPv2. A total of 955,690 SNPs were generated after imputing the missing data points by accepting a 5% mismatch with the closest neighbor in small SNP windows across the entire maize database (~ 22,000 *Zea* samples). The criteria for filtering SNPs for GWAS, PCA (principal component analysis) and LD (linkage disequilibrium) analysis were based on Suwarno et al*.*^20^ with slight modifications. SNPs were filtered based on a call rate (CR) > 0.7 and with a minor allele frequency (MAF) ≥ 0.03, for association analysis and with CR of ≥ 0.9 and MAF of ≥ 0.1% for principal component analysis (PCA).

### Genome-wide association studies (GWAS)

**Figure 1:**
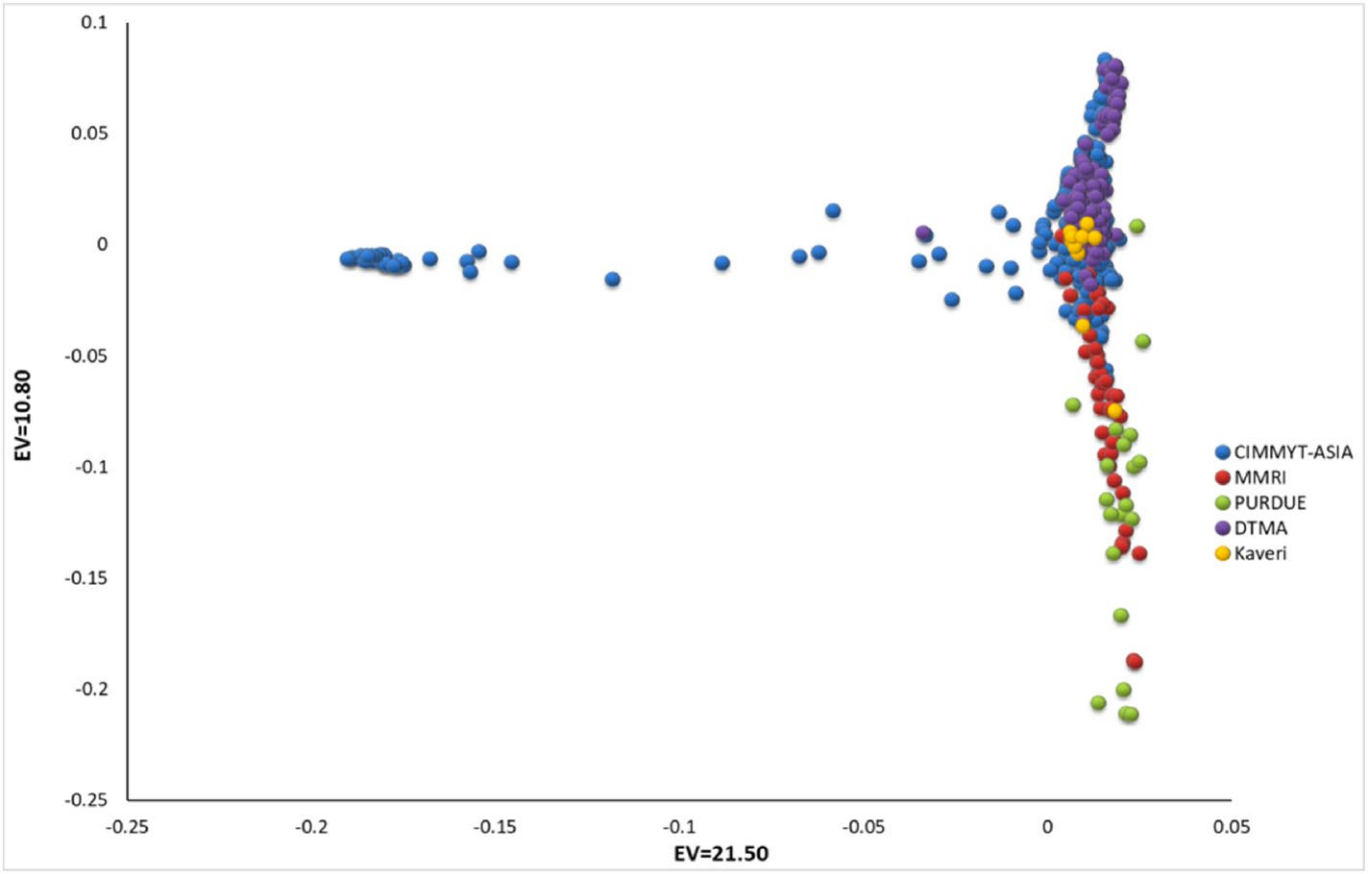
Grouping of accessions of HTAM panel based on first two Principal components.

A total of 955,690 SNPs obtained for the HTAM panel after imputation, were further filtered using the multiple selection criteria (as described above in the HTAM panel genotyping section) and used for GWAS analysis. The association between the filtered SNPs and the trait of interest (GY) was detected by employing a Mixed Linear Model (MLM) in the SNP and Variation Suits v8.6.0 software (GoldenHelix, Inc., Bozeman, MT, www.goldenhelix.com). GWAS was conducted for individual locations by MLM statistics in which the kinship relationship matrix (K) and population structure matrix (Q-based on the first 10 PCAs) were considered to correct for false positive or spurious associations related to familial relatedness. Marker-trait association (MTA) with a *p *value threshold of ≤ − 10^–5^ was considered as significant association. Fitness of the model was determined by observing the Q–Q (quantile–quantile) plot ****(Supple. Figure 1), the plots of observed –log10 *p* values versus expected –log10 *p* values under the null hypothesis that there is no association between the marker and the phenotype.

### Linkage disequilibrium analysis

Linkage disequilibrium was estimated using 43,705 SNPs filtered from the total SNPs with a CR of > 0.9 and MAF of > 0.3. The extent of genome-wide linkage disequilibrium was estimated based on adjacent pairwise r^2^ values and the physical distance among the SNPs^21^ using the ‘nlin’ function in R^22^. Average pairwise distances in which LD decayed at r^2^ = 0.2 and r^2^ = 0.1 were then calculated based on the model given by Hill and Robertson^23^.

### Haplotype trend regression (HTR) analysis

Haplotype trend regression takes one or more blocks of markers and estimates haplotypes for each block of markers which was then regressed on by-sample haplotype probabilities against a dependent variable. Haplotype blocks were detected based on the block-defining algorithm to minimize historical recombination^24^ from significantly associated SNPs identified at each location. Haplotype frequency was estimated using 50 EM iterations, an EM convergence tolerance of 0.0001 and a frequency threshold of 0.01. In addition to the haplotype blocks detected using the algorithm, the single variant SNPs were also included for further analysis. Trend regression analysis of the haplotypes and SNPs was carried out based on a stepwise regression of the grain yield with the pre-estimated haplotypes with forward elimination. The significant haplotypes blocks were finalized based on the Bonferroni p value cutoff of ≤ 10^–2^ and False Discovery Rate (FDR) p value cutoff of ≤ 10^–3^.

## Results

### Performance of the HTAM panel TC under heat stress

Genotypic variation for grain yield was significant at all the locations, with heritability ranging between 0.44 at RA and 0.69 at BG-2 (Table 1). Mean grain yield under heat stress ranged from 2.25 t ha^−1^ at NG to 6.81 t ha^−1^ at JA-2. Maximum grain yield ranged from 3.22 t ha^−1^ (NG) to 8.06 t ha^−1^ (JA-2), whereas minimum grain yield ranged from 1.15 t ha^−1^ (BJ) to 5.42 t ha^−1^ (JA-2) (Table 1). Apart from the lowest grain yield, NG expressed the least variation between maximum and minimum yields in the trial (1.68 t ha^−1^) indicating that this location experienced the most severe heat stress, followed by RA, the second lowest yielding location with narrow variability (1.89 t ha^−1^). Among nine environments, BG-2 was found to be the best location in terms of expressing genotypic variability, with a 4.22 t ha^−1^ difference between maximum and minimum yields at the location with 3.19 t ha^−1^, the mean yield of the trial. The correlation between grain yield at locations ranged from − 0.198 and maximum of 0.390 (Table 2). Notably none of significant R^2^ value are ≥ 0.5. Out of 36 correlations combinations calculated among locations across 2 years, R^2^ values for 25 combinations were significant, including two negative correlations. Among the 19 positively correlated locations, except four combinations—i.e. between NG and BG1, NG with BG2 and HY and BG2 and JA1, all other significant correlations were found between locations with similar VPD regimes during late vegetative and reproductive stage (Supple. Table 2). The two negatively significant correlations were noted between locations with different VPD regimes.

**Table 2:** Correlation between grain yield at different locations under heat stress.

Year	Locations	2013				2014				
		BG1	LU	NG	RA	BJ	BG2	HY	JA1	JA2
2013	BG1	1.000								
	LU	0.102	1.000							
	NG	0.390***	0.290***	1.000						
	RA	0.293***	0.153**	0.351***	1.000					
2014	BJ	0.226***	0.145*	0.197**	0.118*	1.000				
	BG2	0.241***	0.064	0.215***	0.211**	0.016	1.000			
	HY	0.128*	0.065	0.205***	0.124*	0.282***	0.214***	1.000		
	JA1	0.087	0.126*	0.126*	0.090	− 0.118**	0.098*	− 0.010	1.000	
	JA2	0.040	0.198**	0.125*	0.054	− 0.198***	0.043	0.018	0.281***	1.000

***^,^**^,^*Statistical significance at p < 0.10, 0.01 and 0.05.

### GWAS results

**Figure 2:**
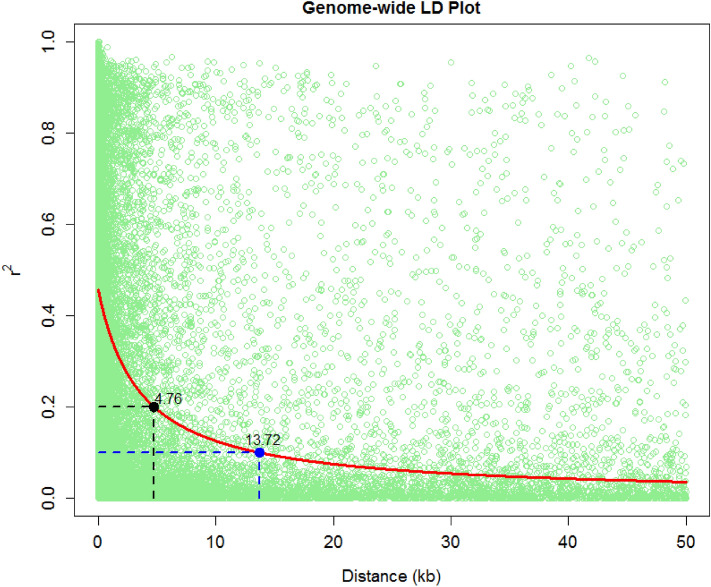
Linkage disequilibrium (LD) decay in the HTAM panel.

**Figure 3:**
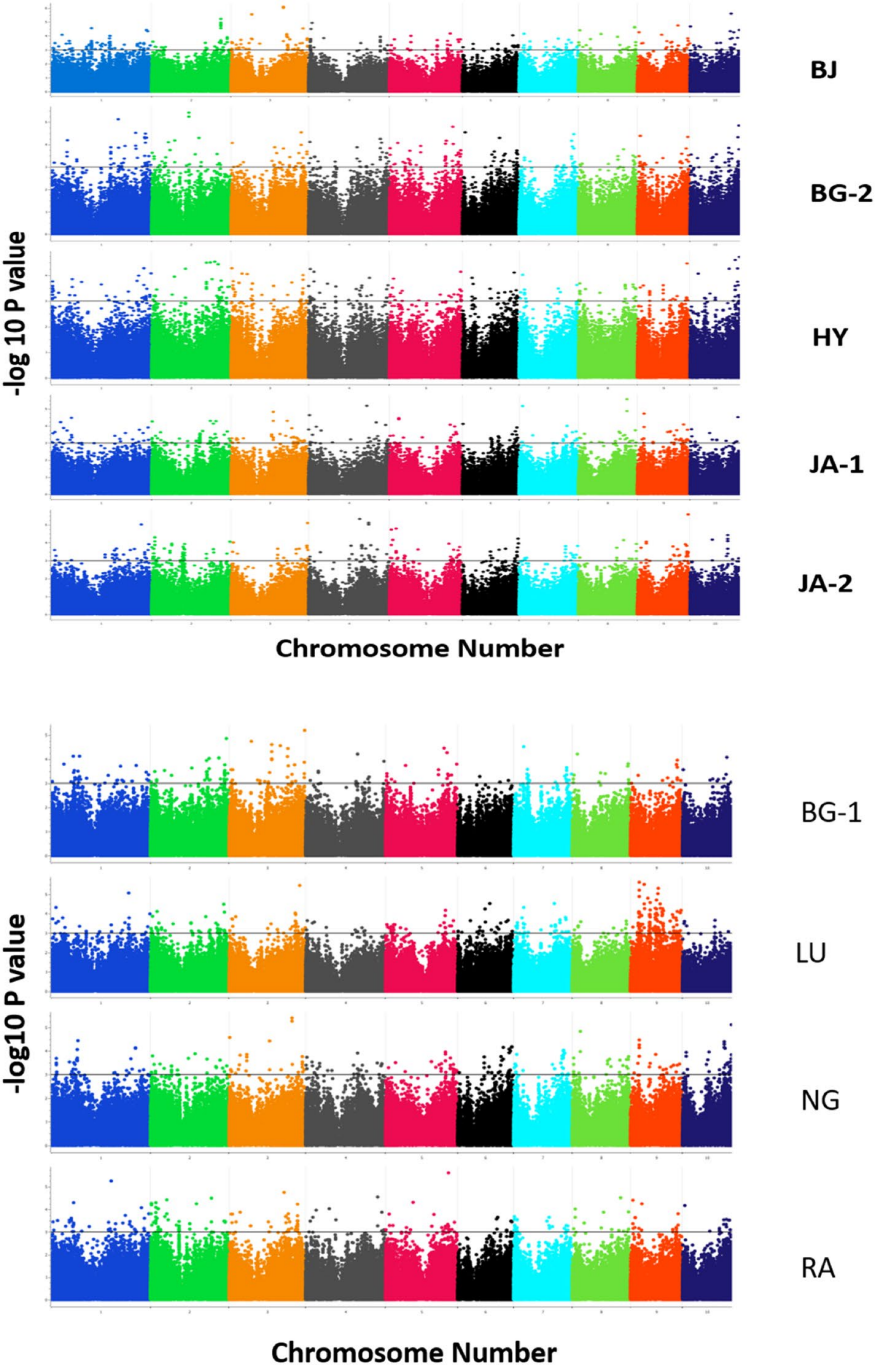
Genome-wide association analysis and significant marker-trait associations for grain yield under heat stress at different phenotyping locations.

In total, 955,690 GBS SNPs were identified for the panel. Principal component analysis using genome-wide markers revealed only a moderate population structure (Fig. 1). The first two components explained 32.30% variance. Upon imposing the selection criteria of a minor allele frequency (MAF) of 0.3 and a call rate of 90%, a sub-set of SNPs was shortlisted to 43,705 SNPs, which were used for Linkage Disequilibrium (LD) decay estimation. The LD decay was 4.76 kb at r^2^ of 0.2 and 13.72 kb at r^2^ of 0.1 (Fig. 2). To carry out the association analysis, the total number of SNPs were filtered based on a call rate of 70% and an MAF of 0.03. The number of SNPs used for GWAS varied from 281,268 SNPs to 289,061 SNPs, as in each location the number of test crosses evaluated varied from 290 to 479 (Table 3). A total of 269 significantly associated SNPs were identified from nine locations at the p value threshold of ≤ 10^–5^ (Fig. 3, Supple. Table 3). The proportion of variation explained ranged from 3.3 to 7.84%, with an average of 4.75%. The number of significantly associated SNPs for each location ranged from 44 in BG-1 to 21 in RA (Table 3). Out of the total significant SNPs, the maximum number of SNPs were identified on chromosome 9 (50 SNPs) and the minimum number of SNPs were in chromosome 7 (12 SNPs) across locations. The p-value threshold of the significantly associated SNPs ranged from 8.08 × 10^–7^ to 9.96 × 10^–5^. Highly significant SNPs were found in the HY location on chromosome 3, viz., S3_157585702, S3_157585709 and S3_157585667. A total of 175 SNPs were found within 140 unique gene models.

**Table 3:** Number of SNPs used for GWAS and PCA analysis and numbers of marker-trait associations (MTA) for grain yield under heat stress at individual locations.

Locations	Number of SNPs used for GWAS	Number of SNPs used for PCA	No of entries tested	Significant MTAs at P ≥ 10^–5^	Significant MTAs at P value cut off*
BG-1	289,060	124,496	290	44	19 (5.02 × 10^–05^)
LU	289,060	124,496	290	29	13 (5.02 × 10^–05^)
NG	289,060	124,496	290	28	9 (5.02 × 10^–05^)
RA	288,826	124,832	335	20	8 (4.66 × 10^–05^)
BJ	281,901	115,936	420	23	9 (4.11 × 10^–05^)
BG-2	289,061	124,496	285	25	12 (4.08 × 10^–05^)
HY	281,268	113,263	479	38	24 (3.96 × 10^–05^)
JA-1	282,186	114,654	471	40	28 (6.09 × 10^–05^)
JA-2	286,786	121,597	435	23	14 (6.05 × 10^–05^)

### Haplotype trend regression (HTR) analysis

In the present study, significant SNPs identified in each location totaled 269 SNPs; they are specific to the location, as none of the SNPs were common across the locations. To make a consensus of the individual location analysis, haplotype trend regression analysis was carried out involving all the 269 SNPs to form the haplotype blocks and regressed against the individual location grain yield. From each location, individual SNP variants or haplotype blocks with bonferroni p value < 10^–2^ and false discovery rate (FDR) p value of < 10^–3^ were selected. The analysis resulted in 26 haplotype blocks in 8 chromosomes (except in chromosomes 1 and 7) and 91 single variant SNPs did not form part of haplotype blocks (Supple. Table 4). The proportion of variance explained by haplotype/SNP variants ranged between 3.10 and 18.24%. Among these, 13 variants were found to be significant in more than one location (Table 4), which included 5 haplotype blocks and 8 single SNP variants. Notably the SNP variant SNP3- S5_24337729 was found to be significant in 5 locations (Table 5), viz., LU, NG, BJ, BG-2 and HY followed by Haplotype Hap-9.1 located at 26 Mb on chromosome 9, which was found to be significant in three locations, viz., BG-1, LU and BJ. All the other variants occurred in at least 2 locations.

**Table 4:** Trend regression analysis using significant SNPs and haplotype blocks for grain yield under heat stress conditions.

Haplotype block/SNPs	Chromosome	Markers used	Number of haplotypes	P-Value	R^2^	Bonferroni P	Favorable alleles
SNP-1	1	S1_177613739	2	1.14E−04	3.68E−02	2.77E−02	G
SNP-2	1	S1_202550281	2	6.06E−07	5.42E−02	1.47E−04	T
Hap_4	4	S4_179804506, S4_179804514	2	1.47E−04	5.42E−02	3.57E−02	AT
SNP-3	5	S5_24337729	2	9.32E−08	7.33E−02	2.26E−05	G
SNP-4	5	S5_5626614	2	2.24E−06	6.17E−02	5.45E−04	A
Hap_6	6	S6_164097936, S6_164097938	2	6.15E−07	1.10E−01	1.50E−04	TT
SNP-5	7	S7_30875396	2	3.82E−06	4.88E−02	9.27E−04	G
SNP-6	9	S9_152043636	2	2.84E−05	4.32E−02	6.90E−03	C
Hap_9.1	9	S9_26578085, S9_26618809	3	1.09E−09	9.16E−02	2.66E−07	TT
Hap_9.2	9	S9_26821739, S9_26821740	2	5.24E−05	6.30E−02	1.27E−02	AG
Hap_9.3	9	S9_41360950, S9_41517742	2	5.68E−05	7.62E−02	1.38E−02	TC
SNP-7	10	S10_127255564	2	6.28E−06	7.37E−02	1.53E−03	T
SNP-8	10	S10_143169509	2	4.17E−05	3.95E−02	1.01E−02	T

**Table 5:** Number of significantly associated SNPs and haplotypes common in two and more locations.

Haplotype block/SNPs	Occurrence in number of locations	BG-1	LU	NG	RA	BJ	BG-2	HY	JA-1	JA-2
SNP-1	2									
SNP-2	2		✓						✓	
Hap_4	2	✓	✓							
SNP-3	5		✓	✓		✓	✓	✓		
SNP-4	2			✓		✓				
Hap_6	2		✓	✓						
SNP-5	2								✓	✓
SNP-6	2					✓	✓			
Hap_9.1	3	✓	✓			✓				
Hap_9.2	2	✓		✓						
Hap_9.3	2	✓	✓							
SNP-7	2			✓						
SNP-8	2		✓							✓

## Discussion

Studies have highlighted the potential advantage of incorporating heat with or without drought tolerance into maize germplasm; this has the potential to offset predicted yield losses in current and future climatic scenarios in South Asia, compared to the current high yielding but heat stress-vulnerable maize varieties that are extensively grown in the lowland tropics^25^. Heat stress tolerance is a relatively new trait added to the product profile of the maize breeding pipeline for the lowland tropics in Asia. A limited success was achieved by breeders over the period by relying on the traditional breeding approaches of selecting tolerant lines based on phenotype, despite the known problems due to genotype x environment interaction, low heritability of grain yield under stress, etc.^26–28^. The integration of new molecular breeding approaches and methodologies has helped to understand the genetic basis of the plant response to stress and make informed selection decisions and introgression of heat tolerance into elite genetic backgrounds^29–31^. In the present study, GWAS was carried out in the HTAM panel phenotyped for grain yield under reproductive stage heat stress at nine locations in South Asia. Heat stress during flowering is known to have a negative impact on pollen viability and silk receptivity^32–35^ and cause a decrease in photosynthesis^36^, which eventually affects grain yield, as evident from the mean and range of grain yield in all locations (Table 1). Grain yield at different locations were weakly correlated as none of the correlation coefficient values were ≥ 0.50 (Table 2) and therefore biologically not meaningful^37^. These weak correlations indicate strong genotype by environment interaction effects under heat stress due to different VPD regimes at Tmax at different locations^12^ that resulted in significantly different phenotypic expression in terms of grain yield under heat stress.

Though the GWAS of the panel was carried out with > 280 K SNPs, accuracy of the association analysis was affected by several genetic and non-genetic factors^38^. LD decay is the key factor among them. In the present HTAM panel, the LD decay was 4.76 kb at r^2^ = 0.2 and 13.72 kb at r^2^ = 0.1. This result is in agreement with various CIMMYT association mapping panels of tropical/sub-tropical germplasm for abiotic and biotic stress and nutritional quality traits^39–41^. The LD decay distance is shorter in tropical and sub-tropical lines than in temperate lines because of their diverse nature and a higher number of rare alleles^42^, which result in obtaining high mapping resolution^17,43^.

In the present study, a total of 269 significantly associated SNPs were identified at p value < 10^–5^ (Fig. 3). Of 269 SNPs, 175 SNPs (i.e., 65.05% of the SNPs) were within the 140 unique gene models. Among the SNPs within the gene models, SNPs S1_283602921, S2_184967723, and S5_182091381 on chromosomes 1, 2 and 5 were in the gene model GRMZM2G065374/Zm00001d034298, and GRMZM2G137426/ Zm00001d017138 coding for bHLH47 and bHLH128 genes, respectively. In general, bHLH (basic-Helix-Loop-Helix) transcription factors (TFs) are involved in regulating various abiotic stresses like drought and salinity, along with biological processes such as plant development, flavonoid biosynthesis, flowering and photosynthesis^44–46^. Similarly, the SNP S5_5626614 on chromosome 5 and SNPs S1_281635911, S3_2629620, S5_191376812 on chromosomes 1, 3 and 5 were within the gene models GRMZM2G386273/Zm00001d013172, GRMZM2G028151/Zm00001d034204, GRMZM2G142179/Zm00001d039324 and GRMZM2G057386/Zm00001d017466 coding for bZIP55, EREB107, EREB184 and EREB50, respectively. The bZIP (basic lucine zipper) and EREB TFs play a critical role in myriad biological functions such as cell elongation, organ and tissue differentiation, root growth, plant senescence and light response^47,48^. Apart from these biological functions, these TFs respond to various abiotic stresses like cold, drought and salt^49^, and enhance the tolerance of plants by altering or reprogramming the various metabolic processes. It has also been reported that bHLH genes interact with bZIP and MYB drought tolerant genes^50,51^. The SNP S3_178992082 on chromosome 3 is within the gene model GRMZM2G449033/Zm00001d042843 and codes for the *na1* (*nana plant1)* gene. Similarly, S9_122089710 on chromosome 9 and S2_1035863 on chromosome 2 are within the gene models GRMZM2G077495/Zm00001d047250 and GRMZM2G064212/Zm00001d001790 that code for PLATZ14 and ATG4 genes, respectively. Reports state that na*1* is an important gene in the brassinosteroid biosynthetic pathway, which plays an important role in internode elongation and sex determination in tassel and silk primordia^52^. Similarly, in maize, the high energy consuming processes are endosperm development and grain filling in which the PLATZ protein interacts with the RNAPIII subunits to regulate the transcription of many transfer RNAs (tRNAs) and 5S ribosomal RNA (5S rRNA)^53^. Autophagy related proteins are the primary route for nutrient recycling in plants and become critical during nitrogen stress and under suboptimal field conditions that severely impact maize productivity.

Of all the candidate genes identified in the present study, 51 SNPs/region were reported in previous GWAS and QTL studies. In the present study eight putative candidate gene regions were identified: two putative candidate gene regions for grain yield under heat stress, two for grain yield under drought stress, two for days to 50% anthesis under heat stress, one each for days to 50% anthesis under drought and combined drought and heat stress reported by Yuan et al.^17^ (Supple. Table 3). In the process of improving grain yield under abiotic stress conditions, stress-adaptive secondary traits with high heritability and good correlation with grain yield under stress could improve the efficiency and success of the breeding program^54^. In the present study, a total of 13 SNPs (Supple. Table 3) were found within the QTL regions of heat stress-related secondary trials like leaf firing (11 SNPs), leaf blotching (2 SNPs) and tassel blast (1 SNP)^55^. Similarly, 16 SNPs (Supple. Table 3) were found within QTL regions for heat susceptibility index (HSI) for grain yield (15 SNPs) and HSI for leaf scorching^56^. Apart from the heat stress studies, few of the SNPs were previously reported for other abiotic stresses like drought and waterlogging stress. The SNP S10_25666798 within the candidate gene GRMZM2G037378/Zm00001d023885 was reported for its association with rooting depth under drought stress^39^, and a total of 20 SNPs were found within the QTLs reported for grain yield under waterlogging stress^57^. SNPs within various gene models, which had biological functions related to stress tolerance mechanisms, and the SNPs that were in previously reported functional and QTL regions can be further validated, re-sequenced to identify causal mutations and the most favorable alleles in order to develop simple PCR-based markers for MAS^58,59^.

The significant SNPs identified in the present study from 9 locations were unique, except for two SNPs, viz., S9_26830815 and S9_26830818, which were significantly associated with the trait in BG-1 and NG. Though none of the other SNPs were common across the locations, most of the SNPs between the locations were in proximity (Supple. Table 3) and considered for haplotype trend regression analysis. The haplotype analysis approach considers the natural dependence that exists between SNPs, which is relevant when considering high-density DNA sequences. Haplotypes were identified and haplotype frequencies were estimated, followed by trend regression analysis across all nine locations. This study helped to identify stable variants significantly associated with grain yield under heat stress across locations. Twenty-six haplotype blocks were identified, and 91 single SNP variants did not form part of any haplotype blocks (Supple. Table 4). Twenty-one haplotype blocks were formed with 2 SNPs, 2 blocks each were formed with 3 and 4 SNPs, and 1 block with 5 SNPs. The haplotype blocks with more than two SNPs in strong LDs are more informative than the biallelic SNPs, because of their multiallelic nature^60^. The higher allelic resolution of identified candidate regions will sustain a more accurate delineation of complex marker–trait correlations. The range and mean of phenotypic variance explained were higher than the single SNP-based GWAS. Also, 67.4% of the haplotypes in the HTR analysis had more PVEs (the range of difference was 0.07–12.22%) than the PVEs based on single SNP-based GWAS. This difference indicates the potential of the HTR to increase the PVEs explained for complex traits like grain yield^61^.

The identified haplotype blocks were found to be significant at a single location to a maximum of five locations. A set of 13 variants (5 haplotype blocks and 8 single SNP variants) were found to be significant for grain yield under heat stress in two to five locations (Table 5). Notably, the single variant SNP_3 (S5_24337729), which was significant across 5 locations, and Hap_6 (S6_164097936, S6_164097938), significant in two locations, were found to be within the intervals of two meta QTLs, MQTL5.3 and MQTL6.2, for grain yield and ASI under drought and optimal conditions, as reported by Semagn et al.^62^. These regions are important, as haplotype-based analysis is useful for fine mapping, marker-assisted selection and precise discovery of new genes responsible for heat tolerance, as well as for overcoming the biallelic limitation of single SNP-based analyses^63,64^. In the present study, the genomic regions identified based on individual location GWAS and haplotype trend regression analysis will be increasingly important in future post-genomic approaches for precise selection of environmentally resilient cultivars and to improve resilience of future cultivars to extreme weather conditions such as heat.

## Conclusion

The present study identified genomic regions based on location-wise GWAS conducted for grain yield under heat stress in nine locations across South Asia, followed by HTRs that are robust and stable across locations with a range of heat stress regimes. Validation of these genomic regions in breeding populations followed by introgression of these common genomic regions into elite genetic backgrounds can be used in developing new generations of maize varieties with stable performance under a range of heat stress environments. The trait-associated markers obtained from this study, in the growing quantity of pan-genome sequence data, can help in the precise selection of resilient cultivars for heat stress. Further, the variants identified in this study for grain yield under heat stress can be used as covariates in prediction models in GS approaches for increasing prediction accuracy.

## Supplementary Information

Supplementary Information 1.

Supplementary Information 2.

## References

[1] FAOSTAT. *Food and Agricultural Organisation of the United Nations (FAO), FAO Statistical Database*. http://faostat.fao.org/site/291/default.aspx (2018).

[2] Ray DK, Gerber JS, MacDonald GK, West PC (2015). Climate variation explains a third of global crop yield variability. Nat. Commun..

[3] Lobell DB, Bänziger M, Magorokosho C, Vivek BS (2011). Nonlinear heat effects on African maize as evidenced by historical yield trials. Nat. Clim. Chang..

[4] Cairns JE (2012). Maize production in a changing climate: Impacts, adaptation, and mitigation strategies. Adv. Agron..

[5] Lobell DB, Burke MB, Tebaldi C, Mastrandrea MD, Falcon WP, Naylor RL (2008). Prioritizing climate change adaptation needs for food security in 2030. Science (80–).

[6] Zaidi PH (2020). Stress-resilient maize for climate-vulnerable ecologies in the Asian tropics. Aust. J. Crop Sci..

[7] Tesfaye K, Zaidi PH, Gbegbelegbe S, Boeber C, Rahut DB, Seetharam K, Erenstein O, Stirling C (2017). Climate change impact and potential benefit of heat-tolerant maize in South Asia. Theor. Appl. Climat..

[8] Lobell DB, Field CB (2007). Global scale climate–crop yield relationships and the impacts of recent warming. Environ. Res. Lett..

[9] Wahid A, Gelani S, Ashraf M, Foolad MR (2007). Heat tolerance in plants: An overview. Environ. Exp. Bot..

[10] Cairns JE, Crossa J, Zaidi PH (2013). Identification of drought, heat, and combined drought and heat tolerant donors in maize. Crop Sci..

[11] Lobell DB, Burke MB (2010). On the use of statistical models to predict crop yield responses to climate change. Agric. For. Meteorol..

[12] Vinayan MT, Zaidi PH, Seetharam K, Das RR, Viswanadh S (2020). Genotype-by-environment interaction effects under heat stress in tropical maize. Agronomy.

[13] Pavani N, Kuchanur PH, Patil A, Arunkumar B, Zaidi PH (2019). Stability analysis of stress-resilient maize (*Zea mays* L.) hybrids across stressed and non-stressed environments. Int. J. Curr. Microbiol. App. Sci..

[14] Kumari S, Shahi JP, Vinayan MT, Zaidi PH, Singh AK, Sinha B (2021). Genome-wide association mapping in maize: Status and prospects. 3 BIOTECH.

[15] Frova C, Sari-Gorla M (1994). Quantitative trait loci (QTLs) for pollen thermotolerance detected in maize. Mol. Gen. Genet..

[16] Reimer R, Benjamin S, Melchinger AE, Schrag TA (2013). Root response to temperature extremes: Association mapping of temperate maize (*Zea mays* L.). Maydica.

[17] Yuan Y, Cairns JE, Babu R, Gowda M, Makumbi D, Magorokosho C, Zhang A, Liu Y, Wang N, Hao Z, San Vicente F, Olsen MS, Prasanna BM, Lu Y, Zhang X (2019). Genome-wide association mapping and genomic prediction analyses reveal the genetic architecture of grain yield and flowering time under drought and heat stress conditions in maize. Front. Plant Sci..

[18] Longmei N, Gill GK, Zaidi PH, Kumar R, Nair SK, Hindu V, Vinayan MT, Vikal Y (2021). Genome wide association mapping for heat tolerance in sub-tropical maize. BMC Genom..

[19] Edmeades GO, Deutsch JA (1994). Development of New Maize Genetic Resources.

[20] Suwarno WB, Pixley KV, Palacios N, Shawn R, Zeaxanthin ZEA (2015). Genome-wide association analysis reveals new targets for carotenoid biofortification in maize. Theor. Appl. Genet..

[21] Remington DL, Thornsberry JM, Matsuoka Y, Wilson LM, Whitt SR, Doebley J (2001). Structure of linkage disequilibrium and phenotypic associations in the maize genome. Proc. Natl. Acad. Sci..

[22] R Core Team, *R: A Language and Environment for Statistical Computing*. R Foundation for Statistical Computing, Vienna, Austria. https://www.R-project.org (2017).

[23] Hill WG, Robertson A (1968). Linkage disequilibrium in finite populations. Theor. Appl. Genet..

[24] Gabriel SB, Schaffner SF, Nguyen H, Moore JM, Roy J, Blumenstiel B (2002). The structure of haplotype blocks in the human genome. Science.

[25] Tesfaye K, Gideon K, Cairns JE, Zaman-Allah M, Wegary D, Zaidi PH, Boote KJ, Rahut DB, Erenstein O (2018). Potential benefits of drought and heat tolerance for adapting maize to climate change in tropical environments. Clim. Risk Manag..

[26] Hussain T, Khan IA, Malik MA, Ali Z (2006). Breeding potential for high temperature tolerance in corn (*Zea mays* L.). Pak. J. Bot..

[27] Rahman SU, Arif M, Hussain K, Hussain S, Mukhtar T, Razaq A, Iqbal RA (2013). Evaluation of maize hybrids for tolerance to high temperature stress in Central Punjab. Am. J. Bioeng. Biotech..

[28] Rahman SU, Arif M, Hussain K, Arshad M, Hussain S, Mukhtar T, Razaq A (2015). Breeding for heat stress tolerance of maize in Pakistan. J. Environ. Agric. Sci..

[29] Xue Y, Warburton ML, Sawkins M, Zhang X, Setter T, Xu Y (2013). Genome-wide association analysis for nine agronomic traits in maize under well-watered and water-stressed conditions. Theor. Appl. Genet..

[30] Thirunavukkarasu N, Hossain F, Arora K, Sharma R, Shiriga K, Mittal S (2014). Functional mechanisms of drought tolerance in subtropical maize (*Zea mays* L.) identified using genome-wide association mapping. BMC Genom..

[31] Matsui T, Omasa K, Horie T (2001). The difference in sterility due to high temperatures during the flowering period among Japonica-Rice varieties. Plant Prod. Sci..

[32] Cicchino M, Edreira JIR, Otegui ME (2010). Heat stress during late vegetative growth of maize: Effects on phenology and assessment of optimum temperature. Crop Sci..

[33] Stone P (2001). The effects of heat stress on cereal yield and quality. Crop Responses Adapt Temp. Stress.

[34] Alam A, Seetharam K, Zaidi PH, Dinesh A, Vinayan MT, Kumar U (2017). Dissecting heat stress tolerance in tropical maize (*Zea mays* L.). Field Crop Res..

[35] Zhi-qiang TAO, Yuan-quan C, Chao LI, Juan-xiu ZOU, Peng Y, Shu-fen Y (2016). The causes and impacts for heat stress in spring maize during grain filling in the North China Plain—a review. J. Integr. Agric..

[36] Flint-Garcia SA, Thornsberry JM, Buckler ES (2003). Structure of linkage disequilibrium in plants. Annu. Rev. Plant Biol..

[37] Skinner DZ, Bauchan GR, Auricht G, Hughes S (1999). Developing a core collection from a large annual *Medicago germplasm* collection. Core Collections for Today and Tomorrow.

[38] Yu J, Buckler ES (2006). Genetic association mapping and genome organization of maize. Curr. Opin. Biotechnol..

[39] Zaidi PH, Seetharam K, Krishna G, Krishnamurthy L, Gajanan S, Babu R (2016). Genomic regions associated with root traits under drought stress in tropical maize (*Zea mays* L.). PLoS One.

[40] Rashid Z, Singh PK, Vemuri H, Zaidi PH, Prasanna BM, Nair SK (2018). Genome-wide association study in Asia-adapted tropical maize reveals novel and explored genomic regions for sorghum downy mildew resistance. Nat. Sci. Rep..

[41] Hindu V, Palacios-Rojas N, Babu R, Suwarno WB, Rashid Z, Usha R (2018). Identification and validation of genomic regions influencing kernel zinc and iron in maize. Theor. Appl. Genet..

[42] Yan J, Shah T, Warburton ML, Buckler ES, McMullen MD, Crouch J (2009). Genetic characterization and linkage disequilibrium estimation of a global maize collection using SNP markers. PLoS One.

[43] Suwarno WB, Pixley KV, Palacios-Rojas N, Kaeppler SM, Babu R (2014). Formation of heterotic groups and understanding genetic effects in a provitamin a biofortified maize breeding program. Crop Sci..

[44] Ito S, Song YH, Josephson-Day AR, Miller RJ, Breton G, Olmstead RG (2012). FLOWERING BHLH transcriptional activators control expression of the photoperiodic flowering regulator CONSTANS in Arabidopsis. Proc. Natl. Acad. Sci..

[45] Babitha KC, Ramu SV, Nataraja KN, Sheshshayee MS, Udayakumar M (2015). EcbZIP60, a basic leucine zipper transcription factor from *Eleusine coracana* L. improves abiotic stress tolerance in tobacco by activating unfolded protein response pathway. Mol. Breed..

[46] Liu Z, Xin M, Qin J, Peng H, Ni Z, Yao Y (2015). Temporal transcriptome profiling reveals expression partitioning of homeologous genes contributing to heat and drought acclimation in wheat (*Triticum aestivum* L.). BMC Plant Biol..

[47] Jakoby M, Wang HY, Reidt W, Weisshaar B, Bauer P (2004). FRU (BHLH029) is required for induction of iron mobilization genes in *Arabidopsis thaliana*. FEBS Lett..

[48] Weiste C, Pedrotti L, Selvanayagam J, Muralidhara P, Fröschel C, Novák O (2017). The Arabidopsis bZIP11 transcription factor links low-energy signalling to auxin-mediated control of primary root growth. PLoS Genet..

[49] Banerjee A, Roychoudhury A (2017). Abscisic-acid-dependent basic leucine zipper (bZIP) transcription factors in plant abiotic stress. Protoplasma.

[50] Brown DE, Rashotte AM, Murphy AS, Normanly J, Tague BW, Peer WA (2001). Flavonoids act as negative regulators of auxin transport in vivo in Arabidopsis. Plant Physiol..

[51] Hartmann U, Sagasser M, Mehrtens F, Stracke R, Weisshaar B (2005). Differential combinatorial interactions of cis-acting elements recognized by R2R3-MYB, BZIP, and BHLH factors control light-responsive and tissue-specific activation of phenylpropanoid biosynthesis genes. Plant Mol. Biol..

[52] Hartwig T, Chuck GS, Fujioka S, Klempien A, Weizbauer R, Potluri DPV (2011). Brassinosteroid control of sex determination in maize. Proc. Natl. Acad. Sci..

[53] Li Q, Wang J, Ye J, Zheng X, Xiang X, Li C (2017). The maize imprinted gene floury3 encodes a PLATZ protein required for tRNA and 5S rRNA transcription through interaction with RNA polymerase III. Plant Cell.

[54] Ziyomo C, Bernardo R (2013). Drought tolerance in maize: Indirect selection through secondary traits versus genomewide selection. Crop Sci..

[55] McNellie JP, Chen J, Li X, Yu J (2018). Genetic mapping of foliar and tassel heat stress tolerance in maize. Crop Sci..

[56] Frey FP, Presterl T, Lecoq P, Orlik A, Stich B (2016). First steps to understand heat tolerance of temperate maize at adult stage: Identification of QTL across multiple environments with connected segregating populations. Theor. Appl. Genet..

[57] Zaidi PH, Rashid Z, Vinayan MT, Almeida GD, Phagna RK, Babu R (2015). QTL Mapping of agronomic waterlogging tolerance using recombinant inbred lines derived from tropical maize (*Zea mays* L.) germplasm. PLoS One.

[58] Harjes CE, Rocheford TR, Bai L, Brutnell TP, Kandianis B, Sowinski SG (2010). Natural Genetic variation in lycopene epsilon cyclase tapped for maize biofortification carlos. Science.

[59] Yan J, Kandianis CB, Harjes CE, Bai L, Kim E-H, Yang X (2010). Rare genetic variation at *Zea mays* crtRB1 increases beta-carotene in maize grain. Nat. Genet..

[60] Stephens JC, Schneider JA, Tanguay DA, Choi J, Acharya T, Stanley SE (2001). Haplotype variation and linkage disequilibrium in 313 human genes. Science (80).

[61] Maldonado C, Mora F, Scapim CA, Coan M (2019). Genome-wide haplotype-based association analysis of key traits of plant lodging and architecture of maize identifies major determinants for leaf angle: hapLA4. PLoS One.

[62] Semagn K, Beyene Y, Warburton ML, Tarekegne A, Mugo S, Meisel B (2013). Meta-analyses of QTL for grain yield and anthesis silking interval in 18 maize populations evaluated under water-stressed and well-watered environments. BMC Genom..

[63] Lesk C, Rowhani P, Ramankutty N (2016). Influence of extreme weather disasters on global crop production. Nature.

[64] Contreras-Soto RI, Mora F, de Oliveira MAR, Higashi W, Scapim CA, Schuster I A (2017) Genome-wide association study for agronomic traits in soybean using SNP markers and SNP-based haplotype analysis. *PLoS One* **12**, e0171105. 10.1371/journal.pone.0171105 PMC5289539 28152092

